# Supplementary material for: Morphological and quantitative analysis of leukocytes in free-living Australian black flying foxes (Pteropus alecto)
Source: PLoS One. 2022 May 25;17(5):e0268549. doi: 10.1371/journal.pone.0268549 (PMC9132326; doi:10.1371/journal.pone.0268549)
Supplement: S1 Table — (DOCX) [file pone.0268549.s001.docx]

**Supporting Information**

**S1 Table. Sampling effort across sites, sexes, and years.**

|  | Redcliffe | | Toowoomba | |
| --- | --- | --- | --- | --- |
|  | Number of Males | Number of Females | Number of Males | Number of Females |
| 2018 | | | | |
| June | 0 | 0 | 0 | 1 |
| July | 2 | 1 | 4 | 2 |
| September | 6 | 7 | 6 | 2 |
| December | 1 | 3 | 2 | 4 |
| 2019 | | | | |
| May | 6 | 2 | 7 | 7 |
| July | 4 | 3 | 8 | 7 |
| September | 4 | 5 | 12 | 11 |
| December | 3 | 3 | 2 | 3 |
| 2020 | | | | |
| July | 0 | 0 | 1 | 7 |
