# Supplementary material for: Morphological and quantitative analysis of leukocytes in free-living Australian black flying foxes (Pteropus alecto)
Source: PLoS One. 2022 May 25;17(5):e0268549. doi: 10.1371/journal.pone.0268549 (PMC9132326; doi:10.1371/journal.pone.0268549)
Supplement: Attachment — Submitted filename: PONE_D2135366_review.pdf [file pone.0268549.s002.pdf]

Morphological and quantitative analysis of hemocytes in free-living Australian black flying foxes (*Pteropus alecto*).
PLoS ONE
PloS ONE/2021/D2135366
Hansen et al.

This paper reports the morphological and descriptive statistics of leukocyte parameters from 134 Bat flying foxes (*Pteropus alecto*) from Australia between 2018-2020. *Pteropus* species are known to be hosts of many zoonotic viruses of interest and therefore a descriptive study of this kind is incredibly important for understanding baseline health of these animals. Overall, this paper does a fantastic job of summarizing key statistics, including differences in animals with injuries and those deemed healthy, and provides a jumping off point for future studies to analyze blood smears. Overall, the paper is written quite well, and I have very few minor comments to improve the paper.

Broad edits/comments:

1. It seems like you took the natural log of eosinophils and monocytes. Perhaps, I missed it but could you provide a short statement on why you did this for these two cell types and not neutrophils and lymphocytes.
2. You mention that you calculate body condition scores in your methods section (line 80). I suggest either include (1) an analysis that explicitly looks at the relationship between body condition and cell counts, (2) create a table that summarizes counts from bats that are in poor (i.e. below a threshold value) or good (i.e. above a threshold value), or (3) that you remove this statement from your methods.

Minor edits:

1. Author list: "Bat One Health" is listed as an author with no affiliation details on the main manuscript document. Is this a mistake?
2. Line 95: As they're anesthetized it likely doesn't matter as much, but could you state when the blood sample was taken? For example, prior to or after the PIT tag insertion.
3. Line 122: Remove the extra space
4. Line 275: Perhaps state what a gametocyte is? (e.g. precursor cells of a parasite)
5. Figure 3 caption: Could you put in the caption which hemoparasite(s) you believe these to be?
