# Supplementary material for: Morphological and quantitative analysis of leukocytes in free-living Australian black flying foxes (Pteropus alecto)
Source: PLoS One. 2022 May 25;17(5):e0268549. doi: 10.1371/journal.pone.0268549 (PMC9132326; doi:10.1371/journal.pone.0268549)
Supplement: Attachment — Submitted filename: Response to Reviewers.docx [file pone.0268549.s004.docx]

Dear Dr. Becker,

**RE: Revisions on manuscript PONE-D-21-35366 'Morphological and quantitative analysis of lymphocytes in free-living Australian black flying foxes (*Pteropus alecto*)'**

We greatly appreciate the time you have taken to review this manuscript. We agree with the edits you have suggested, and after considering the two courses of revision you laid out in your review we have opted to keep the descriptive focus of this paper and remove any inferential language. This had led to changes in the results and discussion sections. Please see lines 176, 242, 272, 282, and 412 for the revisions made in these sections. We have additionally revised our methods section to clarify that we have not performed any formal statistical analyses. Please see lines 141-143.

As a result of the changes made to the discussion section, we are no longer including the following three references:

35. Pacioni C, Robertson ID, Maxwell M, van Weenen J, Wayne AF. Hematologic Characteristics of the woylie (Bettongia penicillata ogilbyi). J Wildl Dis. 2013;49: 816–30.
36. Zuk M, McKean KA. Sex differences in parasite infections: Patterns and processes. Int J Parasitol. 1996;26: 1009–24.
37. Christe P, Glaizot O, Evanno G, Bruyndonckx N, Devevey G, Yannic G, et al. Host sex and ectoparasites choice: preference for, and higher survival on female hosts. J Anim Ecol. 2007;76: 703–10.

To the best of our knowledge, the rest of the reference list is complete and correct, and we cite no retracted articles.

Thank you for your time and valuable comments.

Sincerely,

Dale Hansen, Brooklin E. Hunt, Caylee A. Falvo, Manuel Ruiz-Aravena, Maureen K. Kessler, Jane Hall, Paul Thompson, Karrie Rose, Devin N. Jones, Tamika J. Lunn, Adrienne S. Dale, Alison J. Peel, Raina K. Plowright
